# Spontaneous resolution of asymptomatic hepatic pseudoaneurysm post radiofrequency ablation

**DOI:** 10.1259/bjrcr.20150306

**Published:** 2016-05-20

**Authors:** Li Shyan Ch'ng, Estrellita Elena Mohd Tazuddin, Benny Young, Ahmad Faizal Mohd Ali

**Affiliations:** ^1^ Department of Radiology, Sarawak General Hospital, Kuching, Malaysia; ^2^ Department of Radiology, Universiti Malaysia Sarawak (UNIMAS), Kota Samarahan, Malaysia

## Abstract

Radiofrequency ablation (RFA) of a hepatic tumour is an established treatment option with an acceptable complication rate. Formation of a pseudoaneurysm after RFA of liver metastasis is an uncommon complication. We report the case of a 69-year-old female patient developing a hepatic pseudoaneurysm after RFA of liver metastasis. On a follow-up CT scan 6 weeks later, there was spontaneous resolution of the pseudoaneurysm. Hepatic pseudoaneurysms are usually treated owing to the risk of rupture. Invasive procedures or conservative management of an asymptomatic hepatic pseudoaneurysm is still the subject of debate. The spontaneous resolution of a hepatic pseudoaneurysm in our patient suggests that an asymptomatic pseudoaneurysm maybe observed for resolution instead of being treated at presentation.

## Summary

Radiofrequency ablation (RFA) of a hepatic tumour is a safe procedure with an acceptable morbidity and a low mortality rate. Multicentre surveys show that mortality rates ranged from 0.1% to 0.5%. The major and minor complication rates ranged from 2.2% to 3.1% and 5% to 8.9%, respectively.^1^ Formation of a pseudoaneurysm after RFA of liver metastasis is an uncommon complication. There have been prior reports of symptomatic cases being treated at presentation.^2,3^

## Case report

**Figure 1.**
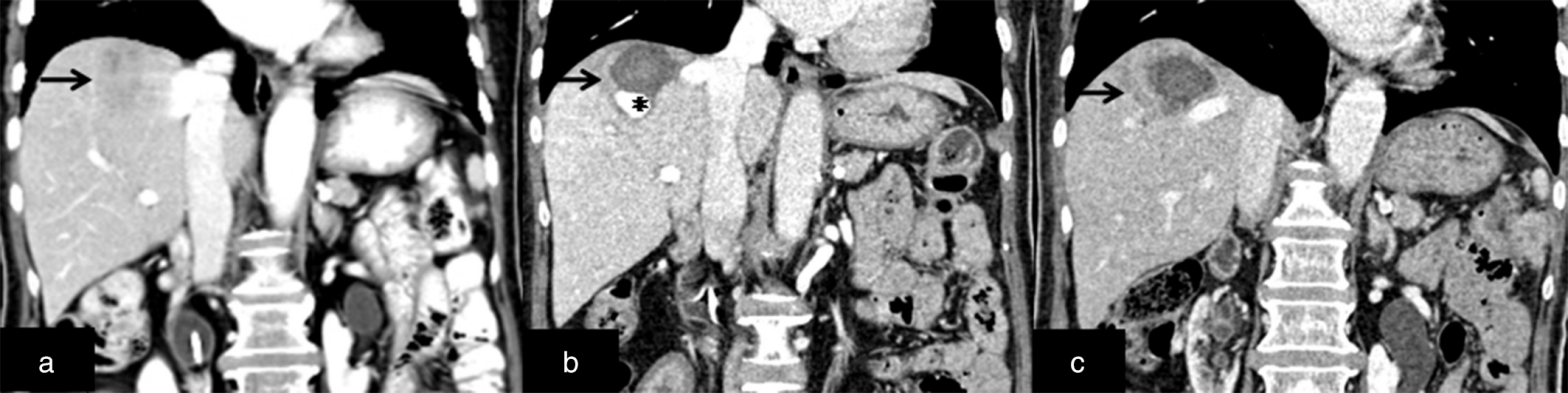
Contrast-enhanced CT scan of the abdomen in the portal venous phase performed at baseline (a), 6 weeks (b) and 12 weeks (c) after the ablation. CT scan at baseline (a) showed a heterogeneously enhancing hypodense metastatic lesion (arrow) in segment VIII. CT scan of the abdomen 6 weeks after ablation (b) revealed residual peripheral tumour with an intensely enhancing area (*) within, measuring 2.3 (width) × 1.4 (AP) cm and suggestive of a pseudoaneurysm. The pseudoaneurysm arises inferolaterally along the electrode pathway. There was resolution of the pseudoaneurysm with increased residual peripheral tumour (arrow) on the CT scan performed 12 weeks after the ablation (c).

A 69-year-old female patient was diagnosed with advanced cervical carcinoma Stage IIIb. She had undergone total abdominal hysterectomy and bilateral salpingo-oophorectomy, as well as completed 36 cycles of radiochemotherapy. A restaging CT scan showed a new solitary segment VIII liver metastasis measuring 3.1 (width) × 3.1 (AP) cm (Figure 1).

In view of the solitary liver metastasis, an RFA was performed. Using the right intercostal approach, an internally cooled 15-cm single electrode with a 3 cm active tip (Cool-tip™, Valleylab, Boulder, CO) was inserted into the tumour’s epicentre under ultrasound guidance. No repositioning of the radiofrequency needle was carried out. Ablation was performed for approximately 12 min. No immediate complication was encountered and the patient was discharged the next day.

A CT scan of the abdomen in the portal venous phase was performed 6 weeks after the ablation. There was residual tumour circumferentially. An intensely enhancing area [measuring 2.3 cm (width) × 1.4 cm (AP)] was noted within the inferolateral aspect of the ablated lesion. The degree of enhancement of the lesion was similar to portal and hepatic veins (Figure 1). No demonstrable communication with the intrahepatic vessels was noted. Based on the CT scan findings, it was thought that the pseudoaneurysm likely originated from the portal or the hepatic vein. However, the single-contrast phase of the CT images made it difficult to identify the origin of the pseudoaneurysm. Extrahepatic disease progression was noted on follow-up CT scan, as evidenced by the enlarged para-aortic nodes and the peritoneal deposit at the splenic hilum. The patient was treated conservatively as she was asymptomatic and not keen on further intervention.

A CT scan of the abdomen 12 weeks after the ablation revealed progression of the segment VIII liver metastasis. The intensely enhancing area was no longer seen, indicating spontaneous resolution of the pseudoaneurysm. There was also progression of other intra-abdominal metastatic disease.

## Discussion

The clinical role of RFA is well established in the treatment of unresectable primary and metastatic hepatic tumours. RFA has been in use since the early 1990s. Complications associated with RFA are rare. The complications reported were abdominal haemorrhage (1.6%), abdominal infection (1.1%), biliary tract damage (1.0%), liver failure (0.8%), pulmonary complication (0.8%), grounding pad burn (0.6%), hepatic vascular damage (0.6%), visceral damage (0.5%), cardiac complication (0.4%), myoglobulinemia or myoglobulinuria (0.2%) and tumour seeding (0.2%).^1,4,5^ Hepatic vascular damage includes portal and hepatic vein thrombosis, hepatic artery damage and pseudoaneurysm.^6^

A pseudoaneurysm may arise from the hepatic artery, or the hepatic or portal vein. The origin of pseudoaneurysms can be identified from multiphase contrast-enhanced CT, ultrasound Doppler or angiography studies. Most commonly, intrahepatic pseudoaneurysms arise from the hepatic artery. The enhancement of the pseudoaneurysm in the case of our patient was similar to the portal and hepatic veins, suggesting that the pseudoaneurysm arose from either the portal or the hepatic vein. Unfortunately, a multiphase contrast-enhanced CT scan was not performed to confirm the origin of the pseudoaneurysm in this patient. Hepatic venous pseudoaneurysms may have a delayed presentation. Park et al^7^ reported a venous pseudoaneurysm that was not apparent on a CT scan performed 4 days after the ablation but was seen on a CT scan performed 3 weeks later.

Pseudoaneurysms after RFA are formed by thermal and mechanical injuries. Thermal injury seldom cause vessel necrosis owing to the cooling effect of blood flow.^8^ The hepatic vein is more susceptible to thermal injury compared with the hepatic artery and the portal vein. This is due to the lack of protective smooth muscle or perivascular connective tissue.^9^ Repeated repositioning of a single needle into the tumour could result in vascular injury. The chances of a mechanical injury occurring from insertion of a single electrode is less than that from a cluster of electrodes.^10^ In our case, we believe that the hepatic pseudoaneurysm was secondary to vessel injury from electrode insertion, as the neck of the aneurysm was along the presumed pathway of the electrode.

Although a pseudoaneurysm is an uncommon complication, it should be kept in mind when reviewing the follow-up CT scans of post-ablation patients.^11^ Rupture rates of hepatic artery aneurysms are as high as 44%, with mortality rates being as high as 82%.^12^ Symptomatic portal vein pseudoaneurysm is usually associated with fistulous communication such as arterioportal shunt,^13^ portobiliary fistula^14^ or portoenteric fistula.^15^ Hepatic pseudoaneurysms can be treated surgically or with minimally invasive techniques such as transcatheter embolization. Minimally invasive procedures have lower mortality and morbidity than surgical intervention.^16^ Embolization can be performed with materials such as coils and *n*-Butyl cyanoacrylate.^3,13,16-19^ A pseudoaneurysm with a wide neck has been successfully treated with a stent graft.^7,20^ Image-guided percutaneous injection of thrombin to treat partially occluded hepatic artery pseudoaneurysms has been reported.^21^ Balloon inflation may be used for temporary occlusion of fistulas prior to surgery.^15^

Thrombosis and resolution of the pseudoaneurysm in our case could be owing to the low pressure of the venous system. Furthermore, the progression of the liver metastasis involving the neck of the aneurysm may have contributed to its resolution. Recurrence of tumour along the margins, as in the case of our patient, can be reduced if two cycles of ablation are performed instead of one or a larger active tip is used. Intrahepatic pseudoaneurysms that resolved 15 days after the RFA were likely due to thrombosis, as reported by Tamai et al.^8^ Tessier et al^16^ reported spontaneous thrombosis of an iatrogenic hepatic artery pseudoaneurysm after 72 months in a patient who refused intervention. Spontaneous thromboses of post-traumatic hepatic artery pseudoaneurysms were noted a few weeks after the trauma in two reported cases.^12,22^ These cases were followed up with serial ultrasound. Pseudoaneurysms are usually treated owing to a high rate of rupture and mortality. The spontaneous resolution of an asymptomatic pseudoaneurysm in our patient and the previously reported cases suggests that not all asymptomatic hepatic pseudoaneurysms need to be treated. Soudack et al^12^ recommend serial ultrasound follow-up of such cases.

## Learning points

1. Hepatic pseudoaneurysm should be kept in mind when reviewing the follow-up CT scans of post-ablation patients owing to a high risk of rupture and mortality.
2. Doppler ultrasound, multiphase contrast-enhanced CT or angiography would be needed to identify the origin of the pseudoaneurysm (venous or arterial origin).
3. A hepatic pseudoaneurysm arising from a vein after RFA is an uncommon complication.
4. The spontaneous resolution of reported asymptomatic pseudoaneurysms suggests that not all hepatic pseudoaneurysms need to be treated. Serial imaging would be needed until resolution of the pseudoaneurysm.

## Consent

Written informed consent was obtained from the patient for publication of this case report, including the accompanying images. This case report has been approved for publication by the local Institutional Review Board.
